# Prognostic Value of Sarcopenia in Metastatic Colorectal Cancer Patients Treated with Trifluridine/Tipiracil

**DOI:** 10.3390/jcm10215107

**Published:** 2021-10-30

**Authors:** Mateusz Malik, Maciej Michalak, Barbara Radecka, Marek Gełej, Aleksandra Jackowska, Emilia Filipczyk-Cisarż, Katarzyna Hetman, Małgorzata Foszczyńska-Kłoda, Beata Kania-Zembaczyńska, Danuta Mańka, Marlena Orlikowska, Hanna Rogowska-Droś, Lubomir Bodnar

**Affiliations:** 1 Lower Silesian Oncology Centre, Clinical Oncology Department, Plac Ludwika Hirszfelda 12, 53-413 Wroclaw, Poland; cisarz.emilia@dco.com.pl; 2 Department of Radiology, Faculty of Medicine, University of Warmia and Mazury in Olsztyn, Aleja Warszawska 30, 11-082 Olsztyn, Poland; macrad1@wp.pl; 3 Diagnostic Imaging Department, MSWiA Hospital, Warmia and Mazury Oncology Centre, Aleja Wojska Polskiego 37, 10-228 Olsztyn, Poland; hanrog@mp.pl; 4 Department of Oncology, Institute of Medical Sciences, University of Opole, Oleska 48, 45-052 Opole, Poland; brad@onkologia.opole.pl (B.R.); mgelej@gmail.com (M.G.); 5 Tadeusz Koszarowski Cancer Center in Opole, Department of Clinical Oncology, Katowicka 66a, 45-061 Opole, Poland; 6 Oncology and Immunooncology Clinic, MSWiA Hospital, Warmia and Mazury Oncology Centre, Aleja Wojska Polskiego 37, 10-228 Olsztyn, Poland; purf_1@wp.pl (A.J.); lubomirbodnar.lb@gmail.com (L.B.); 7 West Pomeranian Oncology Center in Szczecin, Department of Clinical Oncology, Strzalowska 22, 71-730 Szczecin, Poland; khetman@onkologia.szczecin.pl (K.H.); mfoszczynska@onkologia.szczecin.pl (M.F.-K.); 8 Beskid Oncology Centre in Bielsko-Biala, Department of Oncology and Oncohematology, Wyzwolenia 18, 43-300 Bielsko-Biala, Poland; bzembaczynska@gmail.com (B.K.-Z.); danuta.manka@gmail.com (D.M.); 9 Kociewie Health Centre, Oncology Department, Doktora Jozefa Balewskiego 1, 83-200 Starogard Gdanski, Poland; orlik-marlena@o2.pl

**Keywords:** sarcopenia, metastatic colorectal cancer, cancer cachexia, trifluridine/tipiracil

## Abstract

Sarcopenia is common in metastatic colorectal cancer (mCRC), increases the risk of treatment-related toxicity and reduces survival. Trifluridine/tipiracil (TT) chemotherapy significantly improved survival in refractory mCRC patients, but the prognostic and predictive role of pretherapeutic sarcopenia and variation in the skeletal muscle index (SMI) during this treatment has not been investigated so far. In this retrospective, observational study, clinical data on mCRC patients treated with TT at six cancer centres in Poland were collected. Computed tomography (CT) scans acquired at the time of initiation of TT (CT1) and on the first restaging (CT2), were evaluated. SMI was assessed based on the skeletal muscle area (SMA) at the level of the third lumbar vertebra. Progression-free survival (PFS) and overall survival (OS) were calculated from the treatment start. Neither initial sarcopenia nor ≥5% skeletal mass loss (SML) between CT1 and CT2 had a significant effect on PFS in treated patients (*p* = 0.5526 and *p* = 0.1092, respectively). In the multivariate analysis, reduced OS was found in patients with ≥5% SML (HR: 2.03 (1.11–3.72), *p* = 0.0039). We describe the prognostic role of sarcopenia beyond second line treatment and analyze other factors, such as performance status, tumor histological differentiation or carcinoembryonic antigen level that could predict TT treatment response.

## 1. Introduction

Colorectal cancer (CRC) is of considerable epidemiological importance worldwide, as the third most common malignant disease (1.85 million new cases annually; 10.2% of all malignancies). CRC is responsible for approximately 8.9% of cancer-related deaths, with an over 30% increase in the last 15 years [1]. Despite this upward trend in global mortality, 5-year survival in Polish patients with CRC has improved over the past two decades: from 43.3 to 47.6% in men, and from 44.1 to 49.1% in women [2].

Sarcopenia is defined as a loss of skeletal muscle mass associated with reduced muscle strength and impaired physical function. Sarcopenia as a component of cancer cachexia is a complex condition involving nutritional deficiency, chronic inflammation, metabolic imbalance towards hypercatabolism and lower physical activity followed by poor performance status (PS), an increased risk of treatment-related toxicity and reduced survival [3].

Colorectal carcinoma, especially advanced, is itself considered a risk factor for cachexia and sarcopenia. Sarcopenia, both primary (aging-related) and secondary (caused by pathogenic mechanisms), is frequent in patients with CRC, ranging between 12 and 60% in this population [4]. Furthermore, cancer-associated cachexia has been indicated as the pivotal cause of CRC-related deaths, responsible for 22% thereof [5].

Trifluridine and tipiracil hydrochloride (TT) is a combination of a thymidine-based nucleoside analogue, trifluridine and a thymidine phosphorylase inhibitor, tipiracil. The efficacy and safety of TT in patients with metastatic CRC refractory or intolerance to standard therapies were evaluated in the phase III RECOURSE trial [6]. TT is one of the two drugs whose effectiveness has been confirmed in randomized trials. A number of other conventional chemotherapeutic agents, such as capecitabine, mitomycin C and gemcitabine, are used as salvage therapy in CRC, but their beneficial effects in the third- or later-line setting are limited or doubtful. In some patients, retreatment with oxaliplatin or anti-EGFR rechallenge therapy might be an option [7]. Based on this background, TT and regorafenib have been established as standard treatments.

The aim of this study was to assess the incidence of sarcopenia in heavily pretreated metastatic CRC patients treated with TT chemotherapy, and the impact of skeletal muscle loss (SML) on progression-free survival (PFS) and overall survival (OS).

## 2. Materials and Methods

### 2.1. Patients

We retrospectively analyzed 78 patients from six cancer centres in Poland. The main inclusion criteria were as follows: histologically confirmed metastatic or locally advanced, previously treated colorectal cancer; Eastern Cooperative Oncology Group (ECOG) PS of 0, 1 or 2; at least one completed course of TT chemotherapy and availability of the computed tomography (CT) scans acquired as the baseline for the TT treatment (CT1) and as the first control during chemotherapy (CT2), evaluable for the skeletal muscle index (SMI) measurement. The baseline patient characteristics are presented in Table 1. Median age was 64.5 years (30–79), the male/female ratio was 43/35, the colon/rectal cancer ratio was 50/28 and ECOG PS of 0–1/2 was 73/5. All subjects were Caucasian. Prior disease progression during treatment with fluorouracil, oxaliplatin or irinotecan or anti-VEGF therapy and/or anti-EGFR therapy (in case of RAS wild-type status) was a prerequisite before the initiation of the TT treatment. TT was administered at 35 mg/m^2^ of body surface area for 5 days a week (with a 2-day rest) for 2 weeks, followed by a 14-day rest period, which completed each treatment cycle. The course was repeated every 4 weeks. The study was approved by the decision #18/2019/VII of the Warmian-Masurian Medical Chamber Ethics Committee in Olsztyn (Poland).

### 2.2. Image Analysis

**Figure 1:**
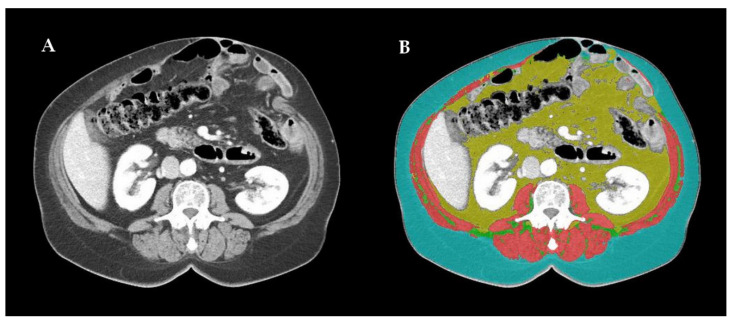
(**A**) Example of contrast-enhanced computed tomography scan of the third lumbar vertebra (L3) region in the venous phase (contains psoas, paraspinal and abdominal wall muscles); (**B**) image processed by Data Analysis Facilitation Suite ver. 2.0.5 by Voronoi Health Analytics, Vancouver, Canada with the Automatic Body composition Analyzer using Computed tomography image Segmentation module provided by Voronoi Health Analytics, Canada (2020); non-optimal segmentations were re-edited using open-source software 3D Slicer ver. 4.11 by 3D Slicer, Boston, MA, USA; skeletal muscles (pink) are separated from intra-abdominal fat (yellow) and subcutaneous fatty tissue (blue).

Imaging-based sarcopenia assessment was carried out by an experienced radiologist using dedicated automatic software: DAFS (Data Analysis Facilitation Suite) ver. 2.0.5 (Voronoi Health Analytics, Vancouver, Canada) with the ABACS (Automatic Body composition Analyzer using Computed tomography image Segmentation) module provided by Voronoi Health Analytics, Canada (2020). Some non-optimal segmentations were re-edited using open-source software 3D Slicer ver. 4.11 (3D Slicer, Boston, Massachusetts, USA). As the third lumbar vertebra (L3) has long been established as a standard landmark, two consecutive CT images, extending at least from L3 to the iliac crest, were chosen to measure the muscle cross-sectional area. Skeletal muscles were identified and quantified based on Hounsfield Unit (HU) thresholds. All the selected CT scans were contrast-enhanced in the venous phase. The L3 region contains psoas, paraspinal and abdominal wall muscles. The L3 SMI was determined as a ratio of the skeletal muscle area (SMA; cm^2^), contoured at the level of the L3 CT scan, to the patient’s height squared (m^2^) [8,9]. An example of this procedure is presented at Figure 1.

### 2.3. Parameters and Statistical Analysis

The L3 SMI below the 5th percentile was chosen as equivalent to sarcopenia, and the sarcopenia cut-off values of 52.4 cm^2^/m^2^ for men and 38.5 cm^2^/m^2^ for women were used for the study population analysis [9]. The predictive and prognostic value of the baseline sarcopenia and subsequent SML (at least 5% decrease in SMI compared to baseline) were analyzed. Progression-free survival (PFS) was calculated from the date of treatment initiation to the date of disease progression defined by the RECIST 1.1 criteria or the date of the last follow-up. Overall survival (OS) was calculated from the date of treatment initiation to the date of death or the final follow-up date. The cut-off date for our analysis was set on 30 November 2020. Univariate analyzes of variables influencing PFS or OS were performed by log-rank test; this identified a preliminary list of significant factors. All the variables found significant or showing a trend towards significance (*p* < 0.1) in the univariate analysis were included in the multivariate analysis. The multivariate analysis of progression-free survival and overall survival were performed by Cox proportional hazards regression model using the forward stepwise method. Medians and life tables were computed using the product-limit estimate by the Kaplan and Meier method, and the log-rank test was employed to assess the statistical significance; *p*-values less than 0.05 were considered to indicate statistical significance. The statistical package MedCalc (ver. 19.7.2; MedCalc Software Ltd., Ostend, Belgium) was used for the analysis.

## 3. Results

### 3.1. Patient Characteristics

Of the initial cohort of 100 patients treated with TT, 78 were eligible for the final analysis; 22 patients were ineligible for lack of CT control or CT without contrast. Patients were enrolled from February 2017 to July 2020. The data cut-off was November 2020. The median observation period was 19.1 months (95% CI, 11.2–19.3), and 66.7% of patients (52/78) had died by the end of follow-up. The average interval between baseline and CT control was 104 days (48–323).

The median SMI, regardless of sex, at CT1 and CT2 was 47.89 cm^2^/m^2^ (27.68–71.95) and 46.43 cm^2^/m^2^ (25.94–70.64), respectively. Based on the assumed SMI thresholds, 34 patients were sarcopenic and 44 were non-sarcopenic at baseline; thus, sarcopenia was initially present in 44% of patients. In both groups the majority of patients did not demonstrate significant (>5%) SMI changes in the follow-up CT, but SMI decrease was more frequent than SMI increase on treatment. Sarcopenia has been reported in 47% of patients at the time of control.

### 3.2. Efficacy

**Figure 2:**
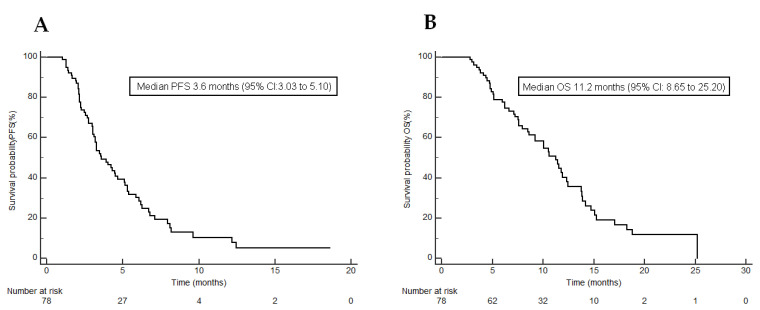
Kaplan–Meier curves showing (**A**) progression-free survival under trifluridine/tipiracil; (**B**) overall survival under trifluridine/tipiracil.

Responses to chemotherapy are presented in Table 2. The Clinical Benefit Rate (CBR) in patients was 47.4% based on the RECIST v.1.1 criteria. Over half of patients (52.6%) did not benefit from the treatment. The median PFS for the entire cohort was 3.6 months (95% CI, 3.03–5.1; Figure 2A).

**Figure 3:**
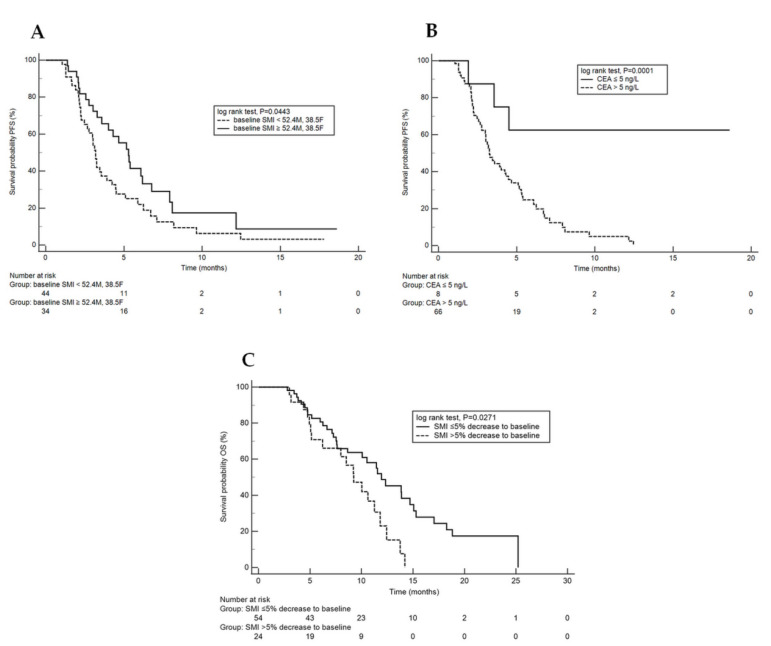
(**A**,**B**): Kaplan–Meier curves (KMC) showing progression-free survival under trifluridine/tipiracil. (**A**): KMC adjusted to baseline sarcopenia (yes or no); (**B**): KMC adjusted to carcinoembryonic antigen (normal or elevated); (**C**): KMC showing overall survival under trifluridine/tipiracil adjusted to SMI significant decrease on treatment (yes or no).

Using the univariate analysis, we found the predictive significance of both baseline SMI and CEA serum level for PFS (*p* = 0.0443 and *p* = 0.0001, respectively) (Figure 3A,B). In the multivariate analysis, we established that an unfavourable independent predictor for TT treatment was high CEA level (>5 ng/L) at baseline (HR: 5.11 (95% CI, 1.56–16.71, *p* = 0.0070)). The results of both univariate and multivariate analyzes of PFS are presented in Table 3 and Table 4.

The median OS for the study population was 11.2 months (95% CI, 8.65–25.20; Figure 2B). The results of the univariate analysis of overall survival indicate that baseline sarcopenia was not associated with poor outcomes in the study cohort (*p* = 0.3963). However, the univariate (Figure 3C) and multivariate analyzes of overall survival revealed that both a >5% decrease in SMI between CT1 and CT2 and poor or unknown histological differentiation achieved the significance levels for predictive factors for reduced survival [HR: 2.03 (1.11–3.72), *p* = 0.0039 and HR: 2.34 (95% CI 1.17–4.66), *p* = 0.0159, respectively; Table 5 and Table 6.

### 3.3. Toxicity

Treatment-related adverse events, according to the National Cancer Institute Common Terminology Criteria for Adverse Events ver. 4.03, occurred in 77 patients (99%), while adverse events of grade 3 or higher occurred in 51 patients (65%; Table 7). The most frequent treatment toxicities were anemia (82%), neutropenia (69%), fatigue (67%), decreased appetite (45%) and weight loss (40%; Table 7). In the study group, 13% of patients required dose reductions, and 94% required dose delay due to drug-related adverse events. No fatal adverse events were reported.

## 4. Discussion

Although sarcopenia has previously been associated with poor prognosis in colorectal cancer, most studies conducted to date have addressed the entire CRC population regardless of the disease advancement or previous treatment. We investigated the association between sarcopenia and PFS/OS in advanced CRC patients treated with TT.

In our study, we identified the unfavourable predictive role of both baseline SMI (<52.4 cm^2^/m^2^ for men and <38.5 cm^2^/m^2^ for women) and CEA serum level (>5 ng/L) for the TT therapy. Although baseline sarcopenia was not associated with poor outcomes in the study cohort, both a >5% decrease in SMI between CT1 and CT2 and poor or unknown histological differentiation had a negative impact on survival.

It is noteworthy that the median PFS for the entire cohort (3.6 months) was higher than in the TT pivotal trial RECOURSE (2.0 months) or the majority of real-world studies [10]. Furthermore, the median OS for the study population (11.2 months) was also significantly higher than in the RECOURSE trial (7.1 months) or other available real-world studies.

A sarcopenia diagnosis is confirmed by the presence of low muscle quantity or quality. Three parameters need to be measured: muscle strength, muscle quantity and physical performance. Total body fat-free mass (FFM) is considered a more effective diagnostic indicator for cancer cachexia than body mass index (BMI), which can be false negative among patients with sarcopenic obesity. Apparently, muscle mass loss is common in cancer patients and does not only occur in underweight individuals. Weight loss has already been associated with reduced median survival [11]. Several hormonal and immunologic factors have been linked to the possible pathophysiological mechanism of muscle atrophy; these include nerve growth factor (NGF), growth hormone, androgens/estrogens deficiency, abnormalities in protein and amino acid metabolism, and inflammatory cytokines (IL-1β, TNF-α and IL-6) or overproduction of parathyroid hormone-related protein [12,13].

To date, there have been only two small retrospective studies investigating the role of the FFM loss in metastatic colorectal cancer treated with TT (or regorafenib). According to a retrospective analysis by Huemer et al., treatment with regorafenib was associated with a statistically significant skeletal muscle loss in later-line treatment, which was not the case with TT. However, subclassification of patients into three groups, namely normal muscle mass, stable sarcopenia and new-onset sarcopenia, at the initiation of third-line therapy permitted discrimination of overall survival, with 1-year overall survival rates of 61, 29 and 16%, respectively (*p* = 0.04) [14]. It is noteworthy that the skeletal muscle loss was significantly higher in patients treated with regorafenib in comparison to patients who received TT also in the sex- and age-adjusted multivariate analysis, as concluded by Hacioglu et al. [15].

Several studies have been published linking sarcopenia to poor outcomes in either non-metastatic or advanced CRC patients [16,17,18,19,20,21]. As mentioned before, the occurrence of baseline sarcopenia was rare in the study population, especially in women. Furthermore, a number of different patterns of ‘metabolic’ response were observed, as presented in Table 8, which implies that cancer-related sarcopenia and cachexia should be always considered a multifactorial phenomenon. It is estimated that only 40 and 20% of CRC patients initially treated for advanced disease worldwide are able to begin the third- and fourth-line treatment, respectively [22]. Our study suggests the complex image of patients treated with oral chemotherapy after average 2.6 previous treatment regimens. Although this population typically does not benefit in terms of progression-free survival, paradoxically it seems to be more predestined for improved overall survival owing to particular ‘immune resistance’ that allows for responses to previous treatment regimens or better performance status in general. Conversely, in the first-line setting, SML at 3 months was associated with poor objective response rate (ORR; *p* < 0.01), and poor progression-free survival (PFS; *p* = 0.03), and it was an independent predictive factor for poor ORR (*p* = 0.01) and PFS (*p* = 0.04) according to a study by Sasaki et al. [21].

The effect of sarcopenia on chemotherapy toxicity among metastatic CRC patients has been evaluated previously [4]. SML has been described as factor associated with chemotherapy grade 3–4 toxicities including severe nausea, vomiting, peripheral neuropathy, neutropenia and anemia. Significant number of patients in our study developed treatment toxicity including 65% of grade 3–4 adverse events (Table 7). The majority of them were blood count disorders: anemia, neutropenia, lymphopenia, and thrombocy-topenia, and could be considered multifactorial: both as a chemotherapy-induced toxicity and as cancer-related complications connected to sarcopenia. In Table 7, we also present differences of the frequency of toxicities by SML (SMI: ≤5% vs. >5% decrease to baseline) which is comparable in both groups. In our study we did not investigate either correlation between SML and blood count disorders, nor the predictive value of neutropenia or lymphopenia. Available literature indicates association of sarcopenia with immune system functions as a valuable research direction.

A relatively small number of patients, CT images performed in six different institutions and multifactoriality of worse patient outcomes were major limitations to our study. Furthermore, in the present study we defined sarcopenia using sex-specific CT L3 SMI cut-offs of 52.4 cm^2^/m^2^ in men and 38.5 cm^2^/m^2^ in women according to Prado et al., while the fifth percentile was considered the cut-off point between low and normal [9]. However, cut-offs for CT L3 SMI vary in the literature, and this variety depends on differences in analyzed populations, such as age, racial structure, socioeconomic status or diseases present [23]. The additional analysis of two other sex-specific cut-offs available for similar population [24,25] was also negative for mPFS and mOS. Moreover, the diagnosis of sarcopenia is more complex than muscle quantity measurement and needs to be confirmed with other parameters, including muscle strength and physical performance, which was not possible in our study due to its retrospective nature. Relatively higher survival results may be related to partially biased patient selection, resulting from the exclusion of subjects without objective response evaluation. Finally, another limitation was adverse event reporting in the source documentation, which was occasionally incomplete, mostly with respect to subjective toxicities, such as nausea or fatigue.

## 5. Conclusions

Sarcopenia is common in advanced CRC patients treated with TT and affects overall survival. CEA serum level before the treatment onset seems to be a significant predictive factor for TT response, but this needs to be confirmed in further studies. TT chemotherapy in third- or later-line should be personalized as far as practicable due to its relatively low effect and frequent adverse events. Determining the optimal drug sequence in later-line chemotherapy of CRC is a significant challenge due to a small number of patients eligible for studies, and searching for valuable predictive factors requires further research.

## Figures and Tables

**Table 1:** Patient characteristics.

Characteristics	*n*	*%*
Enrolled	78	100
Sex		
Male	43	55
Female	35	45
Age, years median (range)	64 (30–78)	
ECOG performance status		
0	22	28
1	51	65
2	5	6
Primary site		
Cecum	7	9
Ascending colon	4	5
Hepatic flexure of the large intestine	3	4
Transverse colon	4	5
Splenic flexure of the large intestine	4	5
Colon descending	3	4
Sigmoid	17	22
Rectosigmoid junction	8	10
Rectum	28	36
Primary tumor location		
Right colon	17	22
Left colon	61	78
Primary tumor histological subtype		
Adenocarcinoma	73	94
Mucinous adenocarcinoma	4	5
Signet ring cell carcinoma	1	1
Histological differentiation:		
Well	10	13
Moderate	51	65
Poorly	6	8
Unknown	11	14
pT category		
pT1	1	1
pT2	7	9
pT3	48	62
pT4	17	22
Not operated on	5	6
pN category		
pN0	18	23
pN1	28	36
pN2	27	35
not operated on	5	6
Site of metastasis		
Liver	49	63
Lung	19	24
Lymph node	18	23
Peritoneum	16	21
*KRAS* mutation status		
Wild-type	36	46
Mutant	40	51
*BRAF* mutation status		
Wild-type	61	78
Mutant	6	7
*NRAS* mutation status		
Wild-type	72	92
Mutant	1	1

Abbreviation: ECOG Eastern Cooperative Oncology Group; pT: size or direct extent of the primary tumor given by histopathologic examination of a surgical specimen according to TNM staging system; pN: degree of spread to regional lymph nodes given by histopathologic examination of a surgical specimen.

**Table 2:** Response in patients by RECIST v.1.1 criteria (*n* = 78).

Tumor Response by RECIST v. 1.1 Criteria	*n*	*%*
CR	0	0
PR	4	5.1
SD	33	42.3
PD	41	52.6
CBR = CR + PR + SD	37	47.4

Abbreviations: CR: complete response; PR: partial response; SD: stable disease; PD: progressive disease; CBR: clinical benefit rate.

**Table 3:** Univariate analysis of progression free survival (log-rank test).

Covariate	*n* (%)	Median (Months)	*p* Value
Age			0.9280
≤70 year	57 (73%)	3.9	
>70 year	21 (27%)	3.3	
Gender			0.1509
Male	43 (55%)	3.5	
Female	35 (45%)	3.9	
Performance status (EGOG)			0. 6813
0–1	73 (94%)	3.6	
2	5 (6%)	3.5	
Sites of metastases:			0.0936
Liver	49 (63%)	3.3	
Other	29 (37%)	4.5	
Sites of metastases			0.6377
Lymph nodes	18 (23%)	3.6	
Other	60 (77%)	3.3	
Sites of metastases:			0.4293
Lung	19 (24%)	3.0	
Other	59 (76%)	4.0	
Primary tumor:			0.2698
left	61 (78%)	3.4	
rigth	17 (22%)	4.3	
Histological differentiation:			0.1615
Well/moderate	61 (78%)	4.0	
Poorly/unknown	17 (22%)	3.0	
KRAS			0.1798
Wild-type	36 (46%)	4.2	
Mutated	40 (51%)	3.2	
NRAS			0.8595
Wild-type	72 (92)	3.6	
mutated	1 (1)	4.5	
BRAF			0.8155
Wild-type	61 (78%)	3.5	
mutated	6 (7%)	2.8	
baseline SMI ≥ 52.4M, 38.5F	34 (44%)	5.3	0.0443 *
baseline SMI < 52.4M, 38.5F	44 (56%)	3.2	
SMI ≤ 5% decrease to baseline	54 (69%)	4.0	0.1092
SMI > 5% decrease to baseline	24 (31%)	3.3	
CEA			0. 0001 *
>5 ng/L	66 (85%)	3.3	
≤5 ng/L	8 (10%)	NR	

Abbreviations: ECOG: Eastern Cooperative Oncology Group, M—male; F—female; SMI—skeletal muscle index; CEA—carcinoembryonic antigen; * statistically significant (*p* < 0.05); NS—not significant; NR—not reached.

**Table 4:** Multivariate analysis of progression free survival.

Parameter	*p*	HR	HR 95% Lower	HR 95% Upper
CEA: >5 ng/L vs. ≤ 5 ng/L	0.0070	5.11	1.56	16.71
KRAS: Mutated vs. Wild-type	0.1883	1.41	0.84	2.36
baseline SMI: ≥52.4M, 38.5F vs. <52.4M, 38.5F	0.1362	0.68	0.40	1.13

Abbreviations: M—male; F—Female; SMI—skeletal muscle index; CEA—carcinoembryonic antigen; HR—hazard ratio; NS—not significant.

**Table 5:** Univariate analysis of overall survival (log-rank test).

Covariate	*n* (%)	Median (Months)	*p* Value
Age			0.1875
≤70 year	57 (73%)	11.4	
>70 year	21 (27%)	7.3	
Gender			0.9625
Male	43 (55%)	11.2	
Female	35 (45%)	9.6	
Performance status (EGOG)			0.5605
0–1	73 (94%)	10.6	
2	5 (6%)	11.4	
Sites of metastases:			0.1823
Liver	49 (63%)	10.2	
Other	29 (37%)	11.9	
Sites of metastases			0.4159
Lymph nodes	18 (23%)	9.2	
Other	60 (77%)	10.8	
Sites of metastases:			0.2640
Lung	19 (24%)	13.7	
Other	59 (76%)	10.5	
Primary tumor:			0.2598
Left	61 (78%)	11.2	
Right	17 (22%)	7.6	
Histological differentiation:			0.0428
Well/moderate	61 (78%)	11.5	
Poorly/unknown	17 (22%)	8.6	
KRAS			0.6737
Wild type	36 (46%)	10.5	
Mutated	40 (51%)	10.6	
NRAS			0.4727
Wild type	72 (92)	10.5	
Mutated	1 (1)	9.5	
BRAF			0.1235
Wild type	61 (78%)	10.6	
Mutated	6 (7%)	7.3	
SMI ≥ baseline 52.4M, 38.5F	34 (44%)	11.1	0.6823
SMI < baseline 52.4M, 38.5F	44 (56%)	10.0	
SMI ≤ 5% decrease to baseline	54 (69%)	11.8	0.0271 *
SMI > 5% decrease to baseline	24 (31%)	9.2	
CEA			0.0300
>5 ng/L	66 (85%)	10.2	
≤5 ng/L	8 (10%)	NR	

Abbreviations: ECOG: Eastern Cooperative Oncology Group; M—male; F—female; SMI—skeletal muscle index; CEA—carcinoembryonic antigen; * statistically significant (*p* < 0.05); NS—not significant; NR—not reached.

**Table 6:** Multivariate analysis of overall survival.

Parameter	*p*	HR	HR 95% Lower	HR 95% Upper
Histological differentiation: well/ moderate vs. poorly/ unknown	0.0159	2.34	1.17	4.66
SMI: ≤5% vs. >5% decrease to baseline	0.0039	2.03	1.11	3.72
CEA: >5 ng/L vs. ≤5 ng/L	0.0959	3.29	0.81	13.34

Abbreviations: M—male; F—female; SMI—skeletal muscle index; CEA—Carcinoembryonic antigen; HR—hazard ratio.

**Table 7:** Frequency of Adverse Events.

	Entire Study Population (*n* = 78)		Frequency of Toxicities by SML			
			SMI ≤ 5% Decrease to Baseline (*n* = 54)		SMI > 5% Decrease to Baseline (*n* = 24)	
Event	Any Grade	Grade ≥ 3	Any Grade	Grade ≥ 3	Any Grade	Grade ≥ 3
Any event—no. (%)	77 (99)	51 (65)	54 (100)	39 (72)	23 (96)	12 (50)
Stomatitis—no. (%)	6 (8)	0	3 (6)	0	3 (13)	0
Hand–foot syndrome—no. (%)	0	0	0	0	0	0
Skin lesions—no. (%)	4 (5)	0	3 (6)	0	1 (4)	0
Diarrhea—no. (%)	19 (24)	1 (1)	16 (30)	1 (1)	3 (13)	0
Pneumonia—no. (%)	5 (6)	0	5 (9)	0	0	0
Nausea—no. (%)	16 (21)	2 (3)	9 (17)	0	7 (29)	2 (8)
Vomiting—no. (%)	8 (10)	2 (3)	3 (6)	0	5 (21)	2 (8)
Anemia—no. (%)	64 (82)	15 (19)	43 (80)	9 (17)	21 (88)	6 (25)
Neutropenia—no. (%)	54 (69)	36 (46)	39 (72)	30 (56)	15 (63)	6 (25)
Lymphopenia—no. (%)	30 (38)	4 (5)	21 (39)	3 (6)	9 (38)	1 (4)
Thrombocytopenia—no. (%)	15 (19)	3 (4)	10 (19)	2 (4)	5 (21)	1 (4)
ALT increased—no. (%)	27 (35)	2 (3)	18 (33)	1 (1)	9 (38)	1 (4)
AST increased—no. (%)	23 (29)	2 (3)	19 (35)	1 (1)	4 (17)	1 (4)
Fatigue—no. (%)	52 (67)	8 (9)	35 (65)	4 (7)	17 (71)	4 (4)
Decreased appetite—no. (%)	35 (45)	2 (3)	22 (41)	0	13 (54)	2 (8)
Constipation—no. (%)	8 (10)	0	6 (11)	0	2 (8)	0
Weight loss—no. (%)	31 (40)	0	17 (31)	0	14 (58)	0

**Table 8:** Patterns of ‘metabolic’ response.

	Normal SMI at Baseline *n* = 44 (56%)	Sarcopenia at Baseline *n* = 34 (44%)
SMI 5% decrease on treatment *n* = 24 (31%)	*n* = 18 (23%)	*n* = 6 (8%)
No significant SMI changes *n* = 48 (62%)	*n* = 23 (29%)	*n* = 26 (33%)
SMI 5% increase on treatment *n* = 6 (7%)	*n* = 3 (4%)	*n* = 2 (3%)

Abbreviations: SMI—skeletal muscle index.

## Data Availability

The data that support the findings of this study are available from the corresponding author, upon reasonable request.
